# Specialized group intervention for compulsive exercise in inpatients with eating disorders: feasibility and preliminary outcomes

**DOI:** 10.1186/s40337-018-0200-8

**Published:** 2018-09-11

**Authors:** Nina Dittmer, Ulrich Voderholzer, Mareike von der Mühlen, Michael Marwitz, Markus Fumi, Claudia Mönch, Katharina Alexandridis, Ulrich Cuntz, Corinna Jacobi, Sandra Schlegl

**Affiliations:** 1 Schoen Clinic Roseneck, Am Roseneck 6, Prien am Chiemsee, Germany; 2 Department of Clinical Psychology and E-Mental-Health, Technische Universität Dresden, Dresden, Germany; 3 Department of Psychiatry and Psychotherapy, University Hospital of Freiburg, Freiburg, Germany; 4 Paracelsus Medical University, Salzburg, Austria; 5 Department of Psychiatry and Psychotherapy, University Hospital of Munich (LMU), Munich, Germany

**Keywords:** Eating disorders, Compulsive exercise, Inpatient treatment, Specialized group intervention, Feasibility

## Abstract

**Background:**

Patients with eating disorders (ED) often suffer from compulsive exercise behavior, which is associated with lower short-term response to treatment and poorer long-term outcome. Evidence-based interventions specifically targeting compulsive exercise behavior have been scarce so far. We developed a manualized group therapeutic approach integrating cognitive-behavioral therapy, exercise therapy and exposure with response management to promote healthy exercise behavior. Our objective was to examine the feasibility and acceptance of this new approach as add-on to regular inpatient treatment in a pilot study. Additionally, we wanted to estimate preliminary effect sizes.

**Methods:**

Thirty-two female, adolescent and adult eating disordered inpatients were recruited. According to the 4th ed. of the Diagnostic and Statistical Manual of Mental Disorders (DSM-IV), twenty-six patients met criteria for Anorexia nervosa (AN), two for Bulimia nervosa and four for eating disorder not otherwise specified. Semi-structured interviews were conducted for qualitative evaluation of feasibility and acceptance of the new intervention. Patients completed the Commitment to Exercise Scale (CES) and the Compulsive Exercise Test (CET) for assessment of compulsive exercise, the Eating Disorder Inventory-2 for assessment of eating disorder pathology, the Beck Depression Inventory-II and Brief Symptom Inventory for assessment of depressive and general psychopathology and the Emotion Regulation Skills Questionnaire for assessment of emotion regulation before the beginning and at the end of the group intervention. Additionally, weight gain was monitored.

**Results:**

Feasibility of our approach was confirmed. All patients reported a high satisfaction with both structure and content of the group. Between pre- and post-intervention, patients showed significant reductions in compulsive exercise (effect size CES: 1.44; effect size CET total: 0.93), drive for thinness (effect size: 0.48), depressive symptoms (effect size: 0.36), general psychopathology (effect size: 0.29) and acceptance of emotions (effect size: − 0.62). Patients with AN also showed significant mean weight gain during the intervention (effect size: − 0.44).

**Conclusions:**

Results of our pilot study indicate that our integrative approach to compulsive exercise in ED patients might represent a promising new therapeutic option. Feasibility and acceptance of the intervention were confirmed. Preliminary effect sizes on most outcomes were promising. As improvements in Body-mass-index, eating disorder and general psychopathology are also to be expected by routine inpatient treatment, a large randomized trial is currently underway to evaluate the efficacy of this new intervention.

## Plain English summary

Patients with eating disorders often suffer from compulsive exercise behavior, which is associated with lower short-term treatment success and poorer long-term outcome. Interventions specifically targeting compulsive exercise behavior are scarce. We developed a structured group therapy to promote healthy exercise behavior by reducing both the compulsive nature and excessive amount of the patients’ exercise behavior. We evaluated our new approach in inpatients with eating disorders in a first small study: All patients reported a high satisfaction with both structure and content of the group. During participation in the group therapy, patients showed strong reductions in compulsive exercise behavior, eating disorder and depressive symptoms. Underweight patients also showed a relevant weight gain. Due to these promising results we are currently evaluating this new therapeutic approach in a large clinical trial.

## Background

Eating disorders (ED) are serious psychiatric disorders often associated with a chronic course and significantly elevated mortality rates [1–3]. A common and distinctive symptom of Anorexia nervosa (AN) known for over 100 years is excessive physical activity despite severe emaciation [4]. Compulsive exercise behavior is observed in 31% to 81% of adolescent and adult patients with AN depending on assessment method, sample and measure used to assess compulsive exercise [4–7].

Several studies have shown compulsive exercise to be associated with longer hospital stays and higher rates of suicidal behavior. It also represents a significant predictor for relapse and chronic course of the eating disorder [8–11]. A review of the existing literature found higher levels of dietary restraint [5, 12], anxiety [13–15] and depression [12, 15] to be consistently associated with compulsive exercise in AN. A high level of physical activity, retrospectively assessed before the onset of the ED during adolescence can also be regarded as potential risk factor for AN [6, 16, 17]. Furthermore, obsessive-compulsive symptoms and AN-subtype may be risk factors for compulsive exercise [12, 15, 18]. In the past years, it has been suggested that hypoleptinemia may represent an underlying endocrinological factor driving hyperactivity in AN [19, 20].

Although examined less frequently compared with AN, between 20% and 57% of patients with Bulimia nervosa (BN) are reported to show compulsive exercise behavior [6, 15, 21]. For BN, “excessive exercise” at a frequency of at least twice per week for three months was explicitly listed as one of several inappropriate compensatory behaviors in the 4th ed. of the Diagnostic and Statistical Manual of Mental Disorders (DSM-IV) [22]. In the 5th ed. of the Diagnostic and Statistical Manual of Mental Disorders (DSM-5) [23], the frequency of “excessive exercise” as a compensatory behavior was reduced to once per week for three months. In both DSM-IV and DSM-5, only the quantitative dimension of this behavior was taken into account.

Despite the importance of the phenomenon, there has so far been no consensus concerning terminology and definition of compulsive exercise: Several studies defined “excessive exercise” based on quantitative features such as exercise frequency, intensity and duration [12, 14, 24, 25]. Other studies specifically emphasized the compulsive, ritualized and uncontrollable quality of “compulsive exercise” [26–28] or even regarded it as culturally rooted variant of Obsessive-compulsive disorder (OCD) [25]. However, a recent Delphi Study by Noetel and colleagues [29] found “compulsive exercise” to be the preferred term for describing the phenomenon. Consensus was reached among the international group of experts that a definition of compulsive exercise should consider both quantitative and qualitative dimensions [29]. Recent studies indicate that compulsive exercise is maintained by a complex interplay of different factors - compulsivity, difficulties in emotion regulation, weight and shape concerns and rigid and perfectionistic personality traits [15, 18, 30, 31].

In our opinion, a comprehensive treatment rationale targeting several of these factors is called for to address the complexity of the behavior including the following:

1. Elements of Cognitive-behavioral therapy (CBT) like psychoeducation and cognitive restructuring are essential in challenging dysfunctional beliefs about exercise, weight and shape [29, 32].
2. Exposure and response prevention strategies are needed to target the compulsive quality of the exercise behavior, as they are considered the first-choice intervention for OCD [32]. Recent recommendations for ED interventions put a special focus on exposure treatment [33, 34], further validating the integration of exposure in a treatment protocol for compulsive exercise.
3. ED patients experience higher levels of emotion intensity, have more difficulties in acceptance of emotions and in emotion regulation and show increased use of dysfunctional emotion regulation strategies compared to healthy controls [35]. Compulsive exercise may serve as emotion regulation strategy similar to food restriction or bulimic behaviors [14, 24, 31, 36]. Training affected patients in new and functional long-term emotion regulation strategies seems paramount.
4. Supporting the normalization of eating behavior by supervised meals is considered a central element of general ED treatment [34, 37, 38]. In analogy, a therapeutic approach for compulsive exercise should include exercise-based elements directly supporting normalization of exercise behavior.

In the 1980s and 1990s, first treatment approaches applied response prevention techniques including one hour of supervised bed rest after meals to reduce compulsive exercise behavior [39]. A 2005 review by Hechler and colleagues [40] showed that unstructured psycho-education, self-monitoring of daily physical activity and cognitive restructuring were the most frequently used treatment approaches.

Recently, two new treatment approaches were developed: Hay and colleagues [41] recently conducted a multicenter randomized controlled trial (RCT) in an outpatient setting evaluating a new CBT-based treatment approach. Schlegel and colleagues [42] developed an exercise-based program for outpatients which is currently being evaluated in an RCT.

According to the rationale outlined above, we consider it essential for a leap forward in the management of compulsive exercise to address several of the maintaining factors like compulsivity, emotion regulation or distorted cognitions as well as the establishment of a healthy exercise behavior simultaneously. A therapeutic approach that corresponds to this demand by comprising CBT-based and exercise-based elements is still missing.

Our team aimed to fill this gap to further improve treatment options for affected patients:

We developed an innovative therapeutic approach for compulsive exercise behavior integrating CBT, exposure and response prevention, emotion regulation techniques and exercise-based elements in one manualized group therapeutic approach. The current study aimed to examine feasibility and acceptance of this new specific approach as add-on to regular inpatient treatment in a pilot study. Additionally we aimed at obtaining pre-post data to estimate preliminary effect sizes of the intervention.

## Methods

### Sample

Our sample consisted of *N* = 32 female adolescent and adult ED inpatients admitted to a large hospital for behavioral medicine in Germany (Schoen Clinic Roseneck; Prien am Chiemsee) between November 2012 and January 2013.

Inclusion criteria were: (1) DSM-IV diagnosis of AN (307.1), BN (307.51) and atypical AN or BN/Eating disorder not otherwise specified (EDNOS) (307.50), (2*)* presence of compulsive exercise, which was defined based on modified DSM-IV criteria for OCD, thereby taking into account qualitative and quantitative dimensions (Table 1), (3) age: 14–45 years, (4) informed written consent and, in case of minors, additional informed written consent by legal guardians.

**Table 1:** Working definition of compulsive exercise

Compulsive exercise	
A. Compulsive exercise as defined by (1) and (2): 1) Repetitive exercise that the person feels driven to perform in response to an obsession or according to rules that must be applied rigidly 2) The exercise is aimed at preventing or reducing distress or at preventing some dreaded event or situation; however the compulsive exercise is clearly excessive	
B. At some point during the course of the disorder the person has recognized that the compulsive exercising is excessive or unreasonable	
C. The compulsive exercise causes marked distress, is time-consuming (takes more than one hour a day), significantly interferes with the person’s normal routine, occupational functioning, usual social activities or relationships or is continued despite medical injury or illness	
Criteria A. + C. are considered obligatory, whereas criterion B. is optional	

Exclusion criteria were: (1) body-mass-index (BMI) < 13 kg/m^2^ at the beginning of the intervention, (2) drug, alcohol or other substance abuse, (3) presence of additional severe psychiatric (i.e. psychotic and bipolar disorders) or neurological diseases (i.e. multiple sclerosis) and suicidality, (4) concurrent treatment for OCD, (5) severe somatic complications prohibiting light to moderate supervised exercise, and (6) marked cognitive impairment due to underweight severe enough to preclude attending and following a 100 min group session. The clinical assessment of cognitive impairment was based on the Association for Methodology and Documentation in Psychiatry (AMDP) System [43]: Patients had to report moderate or severe deficits in comprehension, attention span and short- or long-term memory or these deficits had to be observable by the clinician during the screening procedure.

### The intervention

The intervention represents a combination of routine inpatient treatment and a specific intervention for the promotion of healthy exercise behavior as add-on intervention.

#### Routine treatment

The specialized inpatient treatment for patients with ED consists of a multimodal cognitive-behavioral approach and intense psychiatric and internist treatment. All patients receive individual treatment twice per week, a non-specific problem-solving group treatment three times per week and take part in a manualized, symptom-oriented group intervention for ED patients. Furthermore, all ED patients participate in supervised meals three times per day, meal preparation classes, social skills training and art therapy. Patients can also take part in exercise therapy depending on their weight and physical condition. All underweight patients are required to gain at least 700 g per week, which is monitored by bi-weekly weight checks and visualized on individual weight charts. If no sufficient weight gain is reached, further steps include an increase of food intake, administration of high caloric fluids or – in very severe cases – nasal tube feeding.

#### “Healthy exercise behavior” intervention

In addition to routine treatment, all ED patients who participated in the present study took part in a specific manualized group intervention called “Healthy exercise behavior (HEB)” which specifically targets compulsive exercise behavior. The overarching goal of this intervention is threefold: First, to reduce the excessive quantity of the exercise behavior and reestablish “healthy” exercise behavior, taking into account each patient’s current weight and general health condition. Second, to reduce the compulsive quality of the exercise behavior and establish a more flexible exercise regimen. Third, to re-experience joy, social interaction and relaxation when exercising.

The HEB intervention is manual-based, comprises eight sessions (of 100 min), takes place twice a week and is delivered by a clinical psychologist and a sports therapist. It is conceptualized as closed group for eight to ten patients with sequential sessions. During each session, cognitive-behavioral as well as exercise-based treatment elements complement each other. Between the sessions, patients are required to complete homework tasks (e.g. behavioral analyses or interviews with peers). Group sessions are supplemented by individual graded exposure and response prevention tasks concerning exercise behavior guided by one of the therapists. Table 2 summarizes the content of each group session.

**Table 2:** Content of “Healthy exercise behavior (HEB)” intervention

Symptom-oriented group intervention "Healthy exercise behavior (HEB)"			
Session	Content		
	CBT	Exercise therapy	Self-set goals for the next session
1	Introduction: structure, content and goals of HEB	Trying out different kinds of movement, playful^a^ getting to know each other	
	Reflection of individual compulsive exercise behavior		
2	Risk situations for compulsive exercise	Life-kinetic exercise^b^	
	Behavioral analysis of compulsive exercise	Yoga	
3	Group: exposure rationale and preparation Individually: actual exposures		
	Norms concerning "healthy exercise": differentiation between healthy and compulsive exercise behavior	Partner exercise "walking"	
4	Norms concerning "healthy exercise": differentiation between healthy and compulsive exercise behavior	Playful^a^ movement	
5	"Myths and facts": psychoeducation	Instructed exercise on body perception concerning different body structures	
6	Alternatives for coping with high stress	Trying out short, intense movement intervals for releasing high stress	
	Preparation: “One week with healthy leisure and exercise behavior”		
7	Alternative emotion regulation: emotions as guides for needs	Expressing basic emotions	
8	Review: “One week with healthy leisure and exercise behavior”	Trying out various kinds of exercise focusing on joyful, cooperative activities	
	Conclusion		

*CBT* Cognitive-behavioral therapy; ^a ^Playful: Exploration of movement that focuses on fun and social interaction instead of competition and energy consumption; ^b^ Life-kinetics: Mental training that increases physical and cognitive performance by exercises that impose both physical and cognitive demands

### Procedure

Patients were recruited consecutively during inpatient treatment. Eligible participants answered a number of screening questions covering inclusion and exclusion criteria. Subsequently, they received detailed information on the study and gave informed written consent to participate. For minors, an additional briefing of their legal guardians was provided via telephone. Informed consent forms were sent out to the legal guardians and had to be signed and returned. Outcome measures were compulsive exercise, BMI, ED psychopathology and general psychopathology assessed within three days before the beginning and after termination of HEB group.

Following the intervention, semi-structured interviews were conducted by an independent clinician, where patients were given the opportunity for detailed feedback concerning general set-up, therapeutic value and content of the group therapy.

The study protocol was approved by the ethics committee of the University Hospital of Ludwig Maximilian University Munich (project number: 060–13).

### Measures

#### Measures for assessment of eligibility

Structured Interview for Anorexic and Bulimic Disorders for DSM-IV and ICD-10 (SIAB-EX): The SIAB-EX is a semi-standardized expert rating for the assessment of ED symptoms and frequent additional symptoms. For the current study, the diagnostically relevant questions of the SIAB-EX [44] were employed.

Assessment of compulsive exercise: Compulsive exercise was assessed by modified questions of The Structured Clinical Interview for DSM-IV Axis I Disorders (SCID-I) [45, 46] for OCD (section F: anxiety disorders). The questions of this clinical interview can be found in the Appendix.

#### Feasibility and intervention acceptance

For assessment of feasibility and intervention acceptance, we assessed recruitment and retention rates, number of sessions attended, difficulties in integrating the study into routine inpatient treatment and conducted semi-structured interviews. In these interviews patients first rated their satisfaction with the structure of HEB on a 5-point Likert scale from 1 (= very satisfied) to 5 (= not at all satisfied). They also rated whether they would recommend the group to other affected patients (1 = full recommendation to 5 = no recommendation). Following that, they rated the overall helpfulness of HEB as well as which therapeutic topics they considered to be most helpful (1 = very helpful to 5 = not at all helpful). Patients were also asked what they especially liked about the respective therapeutic element and whether they had suggestions for improvement. Answers to these open questions were recorded and transcribed.

#### Outcome measures for pre-post assessment

##### Commitment to Exercise Scale (CES)

The CES [47, 48] is an eight-item self-report measure frequently used for the assessment of compulsive exercise in patients with ED. It reflects obligatory and pathological aspects of exercise. Cronbach’s α for the overall scale was calculated as 0.82 [48], indicating a good internal consistency. In accordance with Thome and colleagues [49], we used a 4-point Likert scale instead of the original answering format. Cronbach’s α in our sample was 0.88, implying a good internal consistency. Convergent and divergent validity of the measure were shown [48].

##### Compulsive Exercise Test (CET)

The CET ([50]; Schlegl S, Dittmer N, Vierl L, Rauh E, Huber T, Voderholzer U: Validation of the German version of the Compulsive Exercise Test in patients with Eat Disord in preparation) is a self-report inventory that assesses additional aspects of compulsive exercise with five subscales: 1. Avoidance and Rule-driven behavior, 2. Weight Control Exercise, 3. Mood Improvement, 4. Lack of Exercise Enjoyment, and 5. Exercise Rigidity. The inventory consists of 24 items that are answered on a six-point Likert scale ranging from 0 (“never true”) to 5 (“always true”). Cronbach’s α for the overall scale was calculated as 0.85 [50], showing the good internal consistency of the CET.

Cronbach’s α in our sample was 0.87, indicating a good internal consistency. Concurrent and divergent validity of the instrument were shown [50].

##### Eating Disorder Inventory-2 (EDI-2)

The EDI-2 [51–53] is a 91-item multidimensional self-report questionnaire considered as gold standard for the assessment of core dimensions of disordered eating and related symptomatology. It consists of 11 subscales and can be applied for adolescents from age 13 onwards and adults. Cronbach’s α for the overall scale was calculated as 0.96 [52], indicating an excellent internal consistency. In our sample Cronbach’s α was 0.84, showing a good internal consistency. Convergent and divergent validity of the measure were shown [52, 53]. Beyond the three main subscales *Drive for Thinness, Bulimia* and *Body Dissatisfaction* of the EDI-2, we used the subscale *Perfectionism*, as perfectionism is considered an important personality characteristic for maintaining compulsive exercise behavior [31].

##### Beck Depression Inventory-II (BDI-II)

The BDI-II [54, 55] is a self-report inventory that consists of 21 items and is widely used as a screening instrument for the presence and severity of depressive symptoms during the past two weeks. It may be used for adolescents and adults from age 13 onwards. Cronbach’s α for the overall scale was between 0.89 ≤ α ≤ 0.93 in psychiatric samples [55], showing an excellent internal consistency. Cronbach’s α in our sample was 0.90, indicating again an excellent internal consistency. Convergent and divergent validity of the measure were shown [55].

##### Brief Symptom Inventory (BSI)

The BSI [56, 57] represents a 53-item self-report inventory that assesses subjective impairment by nine physical and psychological symptom groups. It can be used for adolescents and adults from age 13 onwards. Cronbach’s α for the overall scale was calculated as 0.92 for adults [56], showing an excellent internal consistency. In our sample Cronbach’s α was 0.96, indicating an excellent internal consistency. Validity of the instrument was shown [56].

##### Emotion Regulation Skills Questionnaire (ERSQ)

The ERSQ [58] represents a 27-item self-report instrument that assesses the situation-adapted interplay of different emotion regulation abilities based on the Adaptive Coping with Emotions Model proposed by Berking [59]. It may be used for adolescents and adults from age 12 onwards [60]. Cronbach’s α for the overall scale was calculated as 0.90, showing an excellent internal consistency [58]. In our sample Cronbach’s α was 0.89, indicating again an excellent internal consistency. Validity of the instrument was shown [58]. Based on the findings of Svaldi and colleagues [35], we were especially interested in changes concerning acceptance of emotions and emotion regulation strategies, so we only used the subscales *Acceptance of Emotions* and *Emotion Regulation* of ERSQ.

### Statistical procedures

Data were analyzed using Statistical Package of Social Science (SPSS) software (Version 20.0) [61]. For all outcomes, paired t-tests were used to assess pre-post changes. Effect sizes (ES) were calculated using the formula: $ES=\frac{M_{pre}-{M}_{post}}{SD_{pre}}$ as recommended for single group pre-post study designs [62]. Here, *Mpre* represents the sample mean of the respective variable before the intervention, *Mpost* the mean of the same variable after the intervention and *SDpre* the standard deviation of *Mpre.* An alpha level of *p*≤ 0.05 was applied to all statistical analyses.

## Results

### Participants

Of the 59 consecutively screened patients, 32 adolescent and adult female ED patients (adolescents: *n* = 9; adults: *n* = 23) were eligible: 26 patients met DSM-IV criteria for AN, two for BN and four for EDNOS. Concerning non-eligible patients, 17 patients did not meet inclusion criteria, three patients denied participation and seven patients were not eligible for other reasons. Clinical and demographic characteristics of the sample are presented in Table 3. Of the enrolled 32 patients, nine dropped out over the course of the study, 23 patients completed the study.

**Table 3:** Clinical and demographic characteristics of the sample

Subtype of eating disorder, n (%)	
AN restrictive	19 (59.4%)
AN binge/purge	7 (21.9%)
BN non-purging	1 (3.1%)
BN purging	1 (3.1%)
Atypical AN and atypical BN/EDNOS	4 (12.5%)
Comorbid diagnoses, n (%)	
OCD	2 (6.3%)
MDD	29 (90.6%)
Age, years	
M (SD)	22.66 (8.25)
BMI at admission, kg/m^2^	
M (SD)	15.41 (2.54)
Previous inpatient treatment, n	
M (SD)	1.44 (1.89)
Length current treatment, weeks	
M (SD)	15.69 (6.49)
Time from admission to participation in HEB, days	
M (SD)	42.03 (31.28)
Time spent with compulsive exercise, hours/day	
M (SD)	4.14 (2.74)

*AN* Anorexia nervosa, *BN* Bulimia nervosa, *EDNOS* Eating disorder not otherwise specified, *HEB* “Healthy exercise behavior” intervention, *OCD* Obsessive-compulsive disorder, *MDD* Major depressive Disorder, *BMI* Body-mass-index, *M* Mean, *SD* Standard deviation

### Feasibility and intervention acceptance

**Fig. 1:**
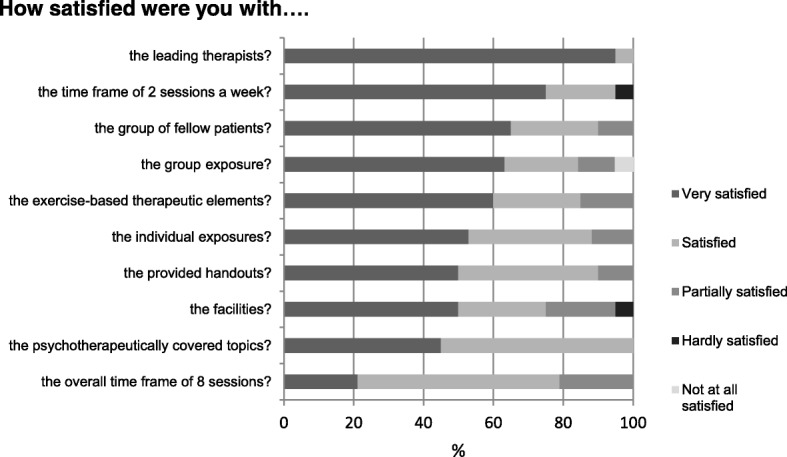
Satisfaction with structure of HEB

Recruitment rate was 54.2%, drop-out rate was 28.1%. However, only two patients (6.2%) specifically withdrew from HEB study while continuing inpatient treatment, the remaining seven patients dropped out of the entire inpatient treatment. All patients who completed the study attended seven to eight HEB sessions. The only difficulty in conducting the study in our inpatient setting was to find time slots where overlap with other treatment elements was minimized. Patients’ satisfaction with the structure of HEB is summarized in Fig. 1.

Regarding recommendation of the intervention to other affected patients, 85% of the patients stated that they “fully recommend” participation in HEB, 15% stated that they would “strongly recommend” participation.Concerning overall helpfulness of the intervention, 40% of the patients indicated that they regarded HEB as “very helpful”, 45% as “pretty helpful” and 15% as “partially helpful”. One patient stated after her participation that *“I made a lot of progress. Of course, I am not completely free of it, but that compulsion is much less. I have to go for a walk, I have to go running - those urges are gone.”*

**Fig. 2:**
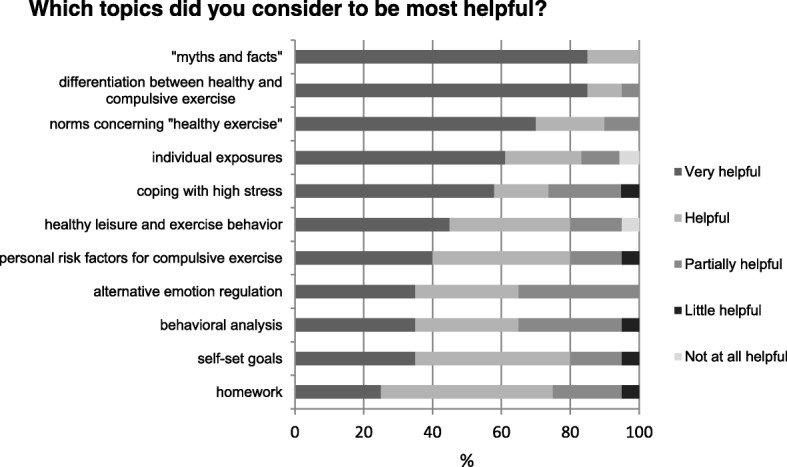
Helpfulness of HEB topics

Figure 2 shows in detail, how the different therapeutic topics covered in HEB were evaluated. Patients especially appreciated the differentiation between healthy and compulsive exercise and the establishment of a healthy norm concerning exercise, psychoeducation and graded exposures. One patient described her astonishment “*that she [her healthy interview partner] does not exercise as much as I thought a normal person would to stay slim.”,* followed by the insight *“And yeah, I really, extremely overdo it.”*

Concerning graded exposures one patient pointedly described *“Yes, to really face my fears in this moment: fears of insane weight gain, fears of what is going to happen if I stop running. And to deal with the topics that arise if I do not run away all the time, literally run away….”*

### Pre-post data

Pre-post changes in compulsive exercise behavior, weight and BMI, ED symptomatology, depressive symptoms, general psychopathology and emotion regulation as well as estimated ES are summarized in Table 4. Patients showed significant reductions in compulsive exercise behavior (effect size CES: 1.44; effect size CET total: 0.93), Drive for Thinness (effect size: 0.48), depressive symptoms (effect size: 0.36), general psychopathology (effect size: 0.29) and Acceptance of emotions (effect size: 0.62). Patients with AN also showed significant mean weight gain during the intervention (effect size: − 0.44).

**Table 4:** Results of quantitative measures

Measure	Pre-Intervention	Post-Intervention	t	*p*	Effect size
	Mean (SD)	Mean (SD)			
Commitment to Exercise Scale	3.23 (0.55)	2.45 (0.56)	8.00	< 0.001	1.44
Compulsive Exercise Test					
Avoidance and Rule-driven Behavior	3.75 (1.06)	2.64 (0.98)	6.76	< 0.001	1.04
Weight Control Exercise	3.29 (1.24)	2.65 (1.07)	4.17	< 0.001	0.52
Mood Improvement	4.29 (0.78)	3.33 (1.09)	5.17	< 0.001	1.23
Lack of Exercise Enjoyment	1.71 (1.27)	1.92 (1.12)	−1.25	n.s.	
Exercise Rigidity	3.83 (1.06)	2.82 (1.03)	4.26	< 0.001	0.95
Total score	16.98 (3.88)	13.39 (3.43)	5.70	< 0.001	0.93
BMI (kg/m^2^) of AN patients	15.67 (1.54)	16.35 (1.50)	−5.91	< 0.001	−0.44
Eating Disorder Inventory-2					
Drive for Thinness	12.81 (6.52)	9.67 (6.36)	5.62	< 0.001	0.48
Bulimia	0.98 (1.71)	0.54 (1.44)	1.65	n.s.	
Body Dissatisfaction	16.83 (7.16)	15.96 (7.18)	1.94	n.s.	
Perfectionism	8.23 (4.44)	6.83 (3.07)	2.37	0.027	0.26
Beck Depression Inventory-II	1.36 (0.55)	1.16 (0.55)	3.42	0.002	0.36
Brief Symptom Inventory (General Severity Index)	1.37 (0.65)	1.18 (0.61)	2.42	0.024	0.29
Emotion Regulation Skills Questionnaire					
Acceptance of Emotions	1.71 (0.89)	2.26 (0.79)	−3.47	0.002	−0.62
Regulation of Emotions	1.40 (0.82)	1.49 (0.79)	−0.68	n.s.	

*BMI* Body-mass-index, *M* Mean, *SD* Standard deviation

## Discussion

The aim of the present pilot study was to examine feasibility and acceptance of a newly developed, add-on intervention to inpatient treatment for compulsive exercise behavior in patients with ED. Additionally we aimed at obtaining preliminary pre-post data to estimate effect sizes of the intervention in preparation for a larger RCT to test the efficacy of the intervention. Our comprehensive approach integrating cognitive-behavioral, exercise-based and exposure and response prevention elements shows promising results:

Implementation of the HEB study proved to be feasible, recruitment and retention rates were comparable to large studies [63], and patients reported good satisfaction with both structure and therapeutic topics of the HEB intervention in qualitative interviews. All patients stated that they would recommend participation in the intervention to other affected patients, indicating a very high acceptance of the treatment protocol.

Patients showed significant reductions on the CES reflecting obligatory and pathological aspects of exercise. We found significant reductions with mostly high effect sizes on most CET subscales. Reductions were especially marked on those subscales which best reflect the compulsive nature of the exercise behavior (CET subscales *Avoidance and Rule-driven Behavior* and *Exercise Rigidity*). Additionally, we observed a significant reduction on the subscale *Mood Improvement,* implying that compulsive exercise was perceived as less enjoyable at the end of the intervention. This might be explained by confrontation with the negative consequences of the compulsive exercise during the HEB intervention. In addition to changes in compulsive exercise behavior, AN patients in our study achieved a significant weight gain. However, weight gain is considered one of the major goals of inpatient treatment, and in the absence of a control group it is unclear which part of the comprehensive intervention primarily drove the weight gain. Scores on patients’ EDI-2 subscales *Drive for Thinness and Perfectionism* were reduced. The significant reductions in these two subscales are of particular importance, as eating pathology and perfectionism are considered key correlates of compulsive exercise in the maintenance model of compulsive exercise behavior proposed by Meyer and colleagues [31]. Over the four weeks of participation in the HEB intervention, patients’ ability to *accept* unpleasant *emotions* when necessary increased. According to the Adaptive Coping with Emotions Model, the ability to accept undesired emotions represents one of the most relevant factors for mental health [59, 64], which highlights the importance of improvement during treatment. Since patients participated in a multimodal therapeutic approach in our hospital, their improved emotion regulation strategies could be attributed to their participation in the HEB intervention directly or possibly to other elements of our approach.

Discussing our results in the light of current research on the topic, the scarcity of the same has to be kept in mind. Schlegel et al. [42] conducted a pilot study to evaluate their exercise-based program and reported high reductions in CES total score together with a dropout rate of 34%. A direct comparison of results seems difficult due to different therapeutic settings (inpatient vs. outpatient) and samples (adults + adolescents with a minimum BMI of 13 kg/m^2^ vs. adults only with a minimum BMI of 17 kg/m^2^). At present, they conduct an RCT for evaluation of the efficacy of their approach. To our knowledge, results of the RCT of Hay and colleagues [41] have not been published yet.

Our concept of HEB seems very much in line with the results of the Delphi study conducted by Noetel and colleagues [29]: Experts recommended to gradually reintroduce healthy exercise under supervision rather than to completely prohibit exercise. Consensus was also reached regarding the importance of psychoeducation on exercise as well as patients learning emotion regulation strategies, identifying risk situations and conducting behavioral analyses for compulsive exercise behavior - all core elements of HEB. For future research, is seems crucial to develop and validate a comprehensive clinical interview for assessing compulsive exercise - which does not exist so far - based on the items where consensus was achieved in the Delphi Study. Due to its pilot character there are several limitations to our study: First, the sample size of our pilot study was small. Second, generalization of our results to the general ED population might be limited by the following two factors: Admission to inpatient treatment implies a considerable severity of eating disorder symptomatology, a BMI < 15 kg/m^2^, somatic complications or continuous weight loss. In addition, ED patients choosing to participate in our study were possibly more motivated and less anxious and rigid concerning the modification of their exercise behavior. Third, as no control group was implemented in our study, our results, especially those concerning BMI increase, have to be interpreted carefully, as all AN patients, irrespective of their participation in our study, were expected to gain at least 0.7 kg per week as part of routine inpatient treatment. Comparable uncontrolled repeated measures study designs were however used in inpatient ED studies by Gale and colleagues [65] as well as Tchanturia and colleagues [66, 67]. Fourth, we did not measure actual levels of exercise by accelerometry. However, we consider measures of actual levels of exercise (as assessed by accelerometry) and of cognitions and emotions around exercise (as assessed by CES and CET) equally important: We assume that changes in dysfunctional exercise cognitions and the development of adequate emotion regulation strategies (e.g. “non-exercise” based emotion-regulation) provide the basis for changes in actual levels of exercise. Additionally, levels of exercise are usually restricted during inpatient treatment, so changes in cognitions and emotions around exercise might even better track “real changes” than changes in exercise levels. Of course, we integrated accelerometry in our subsequent RCT.

## Conclusions

Zipfel and colleagues [68] recognized treatment of compulsive exercise as one of the key unmet challenges in the treatment of ED. Our results indicate that our integrative approach to compulsive exercise in ED patients might represent a promising new therapeutic option. This pilot study confirmed the feasibility and acceptance of the intervention. Preliminary effect sizes on most outcomes were promising.

However, to determine the efficacy of the HEB intervention, a larger dismantling trial is needed, comparing the multimodal routine inpatient treatment (treatment as usual (TAU)) with the additional participation in HEB as add-on module (TAU + HEB) and comprising a sufficiently large sample size. Currently, our research group conducts a large randomized trial to evaluate the efficacy of this treatment approach as add-on element to regular inpatient treatment number ( ISRCTN14208852). The development of three new and different approaches in the treatment of compulsive exercise in ED – one CBT-based [41], one exercise-based [42], and one integrative approach – offers fresh opportunities for this so far neglected phenomenon [69]. Each is currently being evaluated in large RCTs by the respective work groups. Comprehensive, evidence-based therapeutic options for compulsive exercise behavior becoming available will represent a milestone for overall optimization of ED treatment.

## Appendix

### Clinical interview for assessment of compulsive exercise (preliminary version)

A1: Do you have to exercise over and over again and can’t resist doing so? What do you have to do?

A2: Why do you have to exercise? What would happen if you did not exercise?

B: Do you exercise more than you should or that makes sense?

C1: What effect does your exercise behavior have on your life?

C2: Does your exercise behavior bother you a lot?

C3: How much time do you spend exercising?

C4: Do you continue exercising when sick or injured?

## Abbreviations

AN: Anorexia nervosa
BDI-II: Beck Depression Inventory-II
BMI: Body-mass-index
BN: Bulimia nervosa
BSI: Brief Symptom Inventory
CBT: Cognitive-behavioral therapy
CES: Commitment to Exercise Scale
CET: Compulsive Exercise Test
DSM-5: 5th ed. of the Diagnostic and Statistical Manual of Mental Disorders
DSM-IV: 4th ed. of the Diagnostic and Statistical Manual of Mental Disorders
ED: Eating disorders
EDI-2: Eating Disorder Inventory-2
EDNOS: Eating disorder not otherwise specified
ES: Effect size
ERSQ: Emotion Regulation Skills Questionnaire
HEB: “Healthy exercise behavior” intervention
M: Mean
MDD: Major depressive disorder
OCD: Obsessive-compulsive disorder
RCT: Randomized controlled trial
SCID-I: The Structured Clinical Interview for DSM-IV Axis I Disorders
SD: Standard deviation
SIAB-EX: Structured Interview for Anorexic and Bulimic Disorders for DSM-IV and ICD-10
SPSS: Statistical Package of Social Science
TAU: Treatment as usual
